# A pragmatic randomised controlled trial of the Welsh National Exercise Referral Scheme: protocol for trial and integrated economic and process evaluation

**DOI:** 10.1186/1471-2458-10-352

**Published:** 2010-06-18

**Authors:** Simon Murphy, Larry Raisanen, Graham Moore, Rhiannon Tudor Edwards, Pat Linck, Nefyn Williams, Nafees Ud Din, Janine Hale, Chris Roberts, Elaine McNaish, Laurence Moore

**Affiliations:** 1 Cardiff Institute of Society and Health, School of Social Sciences, Cardiff University, 1-3 Museum Place, Cardiff, CF10 3BD, UK; 2 Centre for Economics and Policy in Health, Institute of Medical and Social Care Research, Bangor University, College Road, Bangor, LL57 2DG, UK; 3 Dept. of Primary Care and Public Health, School of Medicine, Cardiff University, North Wales Clinical School, Gwefro Unit 6/7, Wrexham,. LL13 7YP, UK; 4 Social Research Division, Welsh Assembly Government, Cathays Park, Cardiff, CF10 3NQ, UK; 5 Department for Public Health and Health Professions, Welsh Assembly Government, Cathays Park, Cardiff, CF10 3NQ, UK

## Abstract

**Background:**

The benefits to health of a physically active lifestyle are well established and there is evidence that a sedentary lifestyle plays a significant role in the onset and progression of chronic disease. Despite a recognised need for effective public health interventions encouraging sedentary people with a medical condition to become more active, there are few rigorous evaluations of their effectiveness. Following NICE guidance, the Welsh national exercise referral scheme was implemented within the context of a pragmatic randomised controlled trial.

**Methods/Design:**

The randomised controlled trial, with nested economic and process evaluations, recruited 2,104 inactive men and women aged 16+ with coronary heart disease (CHD) risk factors and/or mild to moderate depression, anxiety or stress. Participants were recruited from 12 local health boards in Wales and referred directly by health professionals working in a range of health care settings. Consenting participants were randomised to either a 16 week tailored exercise programme run by qualified exercise professionals at community sports centres (intervention), or received an information booklet on physical activity (control). A range of validated measures assessing physical activity, mental health, psycho-social processes and health economics were administered at 6 and 12 months, with the primary 12 month outcome measure being 7 day Physical Activity Recall. The process evaluation explored factors determining the effectiveness or otherwise of the scheme, whilst the economic evaluation determined the relative cost-effectiveness of the scheme in terms of public spending.

**Discussion:**

Evaluation of such a large scale national public health intervention presents methodological challenges in terms of trial design and implementation. This study was facilitated by early collaboration with social research and policy colleagues to develop a rigorous design which included an innovative approach to patient referral and trial recruitment, a comprehensive process evaluation examining intervention delivery and an integrated economic evaluation. This will allow a unique insight into the feasibility, effectiveness and cost effectiveness of a national exercise referral scheme for participants with CHD risk factors or mild to moderate anxiety, depression, or stress and provides a potential model for future policy evaluations.

**Trial registration:**

Current Controlled Trials ISRCTN47680448

## Background

It is widely recognised that regular physical activity is beneficial to both physical and mental health [1]; it is associated with reduced risk of chronic diseases including coronary heart disease (CHD) [2] and improved mental health [3,4]. Despite recommendations that adults should undertake 30 minutes of moderate intensity exercise at least five times per week, only 30 per cent of adults in Wales are active to this level [5]. Whilst a number of population-based approaches to promoting physical activity have been identified, exercise referral schemes (ERS) represent a more targeted approach for specific patient or population subgroups [6]. This provides an opportunity for patients to have direct contact with and receive advice from qualified health and exercise professionals and to access a range of tailored activities to support and enable lifestyle changes [7]. However, despite the growing numbers of ERS around the UK over the past decade, the evidence base for their effectiveness is weak [6,8].

Previous studies of ERS have evaluated interventions of variable content and intensity, recruited different samples with varying inclusion and exclusion criteria, utilised different outcome measures and adopted variable follow up points. A systematic review of ERS identified six randomised controlled trials (RCTs,) from the UK [9]. Three trials compared 10-12 week long courses of gym-based exercise with exercise information leaflets [10-12]; exercise classes in community halls with no intervention [13]; walking scheme with exercise advice [14]; and gym-based exercise with a walking scheme or exercise advice [15]. Five of these reported on the proportion of participants who were moderately active at the end of the intervention and there was a modest but statistically significant improvement in activity with a combined risk ratio of 1.2 (95% CI = 1.06 to 1.35). The pooled number needed to treat (NNT) indicated that 17 sedentary people need to be treated for one to become moderately active [9]. This modest effect was partly explained by poor rates of uptake and adherence to the schemes and a lack of intervention relapse prevention strategies. Despite only modest improvement in activity levels, one RCT found a relative improvement in depression in the exercise group [15] and another found improvement in health-related quality of life [13].

This review was consistent with previous systematic reviews [16,17] in finding that ERS promote exercise in the short-term, but only in certain populations, that more intensive and longer interventions appear to be more effective; and that they may be more effective in improving mental health or quality of life than in changing long-term exercise habits. Only one RCT of an exercise referral scheme has incorporated a thorough health economic evaluation, which concluded that the exercise intervention was more costly but only slightly more effective than advice alone and was unlikely to be cost-effective [15]. However, there was a large degree of cross-arm contamination. Indeed, methodological concerns over the rigour of many of the above studies have led the National Institute for Health and Clinical Excellence (NICE) to conclude that 'Further research, using a controlled research design, is required to determine the impact that exercise referral scheme may have on reducing health inequalities and their effectiveness in increasing physical activity levels in adult populations" [18].

### The Welsh National Exercise Referral Scheme

In Wales, in the early half of the last decade, most local health board (LHB) areas operated ERS, each following different protocols [6]. The development of high quality schemes was identified as a key action area in a number of Welsh Assembly Government documents [19,20] and in 2006, existing good practice was assessed and standardised in Wales-wide protocols [21]. The National Exercise Referral Scheme (NERS) subsequently replaced local schemes and was rolled out in three phases from 2007. Supported by the Welsh Assembly Government working in partnership with Local Authorities, Public Health Wales (formerly the National Public Health Service) and LHBs, the NERS provided funds for a dedicated Exercise Co-ordinator (EC) and a number Exercise Professionals (EP) in each local health board area.

NERS consists of a series of motivational interviewing (MI) [22] consultations with an EP based in a community sports centre and access to a tailored, subsidised 16 week activity programme. To be eligible for NERS, participants must be sedentary (defined as not moderately active for 3 or more times per week or deconditioned through age or inactivity), and have at least one medical condition, covering CHD risk factors, mental health, musculoskeletal, respiratory/pulmonary and neurological conditions (see Table 1 for scheme inclusion criteria). The primary aim of NERS is for participants to achieve 30 minutes of moderate physical activity on at least 5 days per week. Common features of the scheme are detailed below.

**Table 1:** Scheme inclusion/exclusion criteria and trial eligibility

Scheme inclusion criteria	Scheme exclusion criteria	Trial eligibility
The patient must be **sedentary **(defined as not moderately active for 3 or more times per week or deconditioned through age or inactivity), and have at least one of the following medical conditions: **CHD risk factors** • Raised blood pressure more than 140/90 (either) but less than 180/100 (either) • Weight management • BMI greater than 28 • Controlled diabetes • Impaired glucose tolerance • High cholesterol greater than 5.0 • Family history of heart disease or diabetes • Referral from Cardiac Rehabilitation Schemes (only from phase IV) **Mental health** • Mild anxiety, depression or stress **Musculoskeletal** • At risk of Osteoporosis • Arthritis (mild) • Poor mobility • Musculoskeletal pain including back pain **Neurological conditions** • Multiple sclerosis **Respiratory/pulmonary** • Chronic obstructive pulmonary disorder (COPD) - Mild/moderate well controlled (asthma, bronchitis, emphysema) **Other** • Smoker • Chronic fatigue	• Aged 16 or under • Unstable angina • Blood pressure 180/100 (in either) or above and/or uncontrolled or poorly controlled hypertension • Cardio myopathy • Uncontrolled tachycardia • Cardiac arrhythmia • Valvular heart disease • Congenital heart disease • Unexplained dizzy spells • Excessive or unexplained breathlessness on exertion • Uncontrolled or poorly controlled diabetes • Uncontrolled or poorly controlled epilepsy • History of falls or dizzy spells in the last 12 months • Uncontrolled or poorly controlled asthma (severe COPD) • First 12 weeks of pregnancy • Awaiting medical investigation • Aneurysms • Cerebro-vascular disease • Unstable/newly diagnosed angina (within 6 months) • Established coronary heart disease (including myocardial infarction) • Any other uncontrolled condition	The patient must be **sedentary **and have at least one of the following condition: **CHD risk factors** • raised blood pressure more than 140/90 (either) but less than 180/100 (either) • weight management • BMI greater than 28 • controlled diabetes • impaired glucose tolerance • high cholesterol greater than 5.0 • family history of heart disease or diabetes • referral from Cardiac Rehabilitation Scheme (only from phase IV) , and or **Mental health** • mild anxiety, depression or stress

Delivery of the Welsh national exercise referral scheme:

16 week programme of exercise supervised by a qualified EP

• Initial face to face consultation with EP on entry - lifestyle questionnaire, health check (resting heart rate, blood pressure, BMI, and waist circumference), introduction to facilities, MI and goal setting

• Access to one to one exercise instruction and/or group exercise classes

• Discounted rate for exercise activities £1 per session.

• Four week telephone consultation with EP - review of goals and MI

• Sixteen week face to face consultation with EP - review of goals, MI, health check, lifestyle questionnaire, service evaluation questionnaire [23] and signposted to exit routes

Post 16 week activities

• Range and cost of exit routes dependent on area

• 8 months contact by phone to check progress

• 12 months face to face review including Chester fitness step test [24].

Consultations occur at entry, 4 weeks (by phone) and 16 weeks. Following this, participants are contacted by telephone at 8 months to monitor progress and at 12 months they are invited to attend a review session. Routine programme monitoring systems are maintained by EPs and capture the dates of and records from initial, 4 and 16 week and 8 and 12 month consultations.

The Welsh Assembly Government commissioned an independent evaluation of the scheme as it was implemented in 13 of the 22 LHBs in Wales during phase one. This evaluation was to focus on the effectiveness of the scheme among two priority patient groups, those referred for mental health reasons (anxiety and depression) or for CHD risk factors This paper reports upon the design of the national evaluation, which utilised a randomised controlled trial design with nested process and economic evaluations, and reflects on issues in the development and implementation of the evaluation design for current challenges in policy evaluation [25,26]. Ethical approval for the study was obtained from the Thames Valley Multi-centre Research Ethics Committee (Ref: 06/MRE12/85). Approval from medical directors within each LHB was also obtained.

## Methods and Design

### Study Design

**Figure 1:**
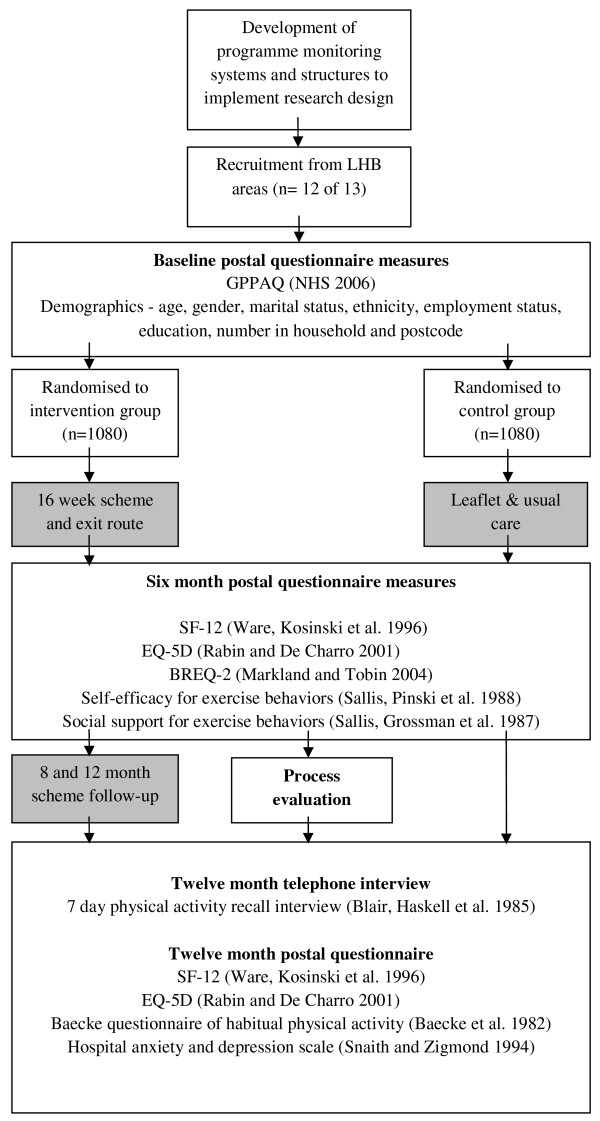
Study Design.

The evaluation study comprised three key components. In order to evaluate the overall effectiveness of the intervention, a randomised controlled design was employed. Mixed methodology RCTs have the potential to provide unbiased estimates of the effectiveness of interventions as well as identifying contextual influences on intervention delivery [27]. A nested process evaluation therefore examined how the initiative was implemented, gained a more in-depth understanding of the views of providers and users, and facilitated interpretation of outcome effects. A nested economic evaluation was also undertaken in the form of a cost utility analysis which measured costs and benefits from a public sector multi-agency perspective, with results expressed as an incremental cost per quality adjusted life year (QALY) gained. In this way, the evaluation not only addresses the question, 'Does it work?', but also considers 'What works?', 'For whom?' 'Under what circumstances?' and 'At what cost?'[28]. A summary of the study design is presented in Figure 1.

### Recruitment

Participants were recruited using opportunistic referral by a range of health professionals. NERS coordinators in each area undertook briefings with primary care teams to explain the trial and to provide referral forms and trial information materials. Health professionals who chose to participate in trial recruitment identified suitable sedentary patients who met scheme inclusion criteria (see Table 1) as part of their normal clinical practice. Patients were provided with basic trial information and a referral form was forwarded to the evaluation team, who assessed trial eligibility criteria (see Table 1). For patients referred for mental health and/or CHD reasons, NERS was only available to those who were willing to participate in the trial. Patients referred for reasons other than mental health and/or CHD were able to access the scheme without participating in the trial and their referral from were forwarded to ECs for processing.

Referred patients who were eligible for the trial were sent full informed consent materials and a brief baseline questionnaire for them to return by post. Recruitment If patients did not want to participate in the trial, they could gain access to the scheme by joining a 12 month waiting list.

### Randomisation

Those consenting to participate in the trial were randomly assigned to an intervention (exercise referral scheme) or control trial arm using a random number generator, with gender and LHB as stratification variables. Randomisation of forwarded referral forms occurred every 2 weeks, with treatment allocation blind and remote from participants and practitioners. The control group received usual health care and a leaflet highlighting the benefits of exercise and including a website address listing local leisure facilities. Control participants were offered priority access to NERS at 12 months.

### Measures

#### Pre randomisation baseline measures

At baseline, participants completed a short postal questionnaire assessing age, gender, marital status, ethnicity, employment status, education, car ownership, number in household, willingness to pay for the scheme and visits to the GP in last 6 months. It also included the General Practice Physical Activity Questionnaire (GPPAQ) [29], which provides a patient activity index which indicates whether the respondent is inactive, moderately inactive, moderately active, or active. Participant post codes were also used to obtain the Welsh Index of Multiple Deprivation [30] of the lower layer super output area in which they lived, with higher scores representing greater deprivation.

#### Outcomes at 12 months

##### - Minutes of weekly activity

The primary outcome is total minutes of weekly activity at 12 months, using the seven day physical activity recall questionnaire (7-DAY PAR) [31]. This interview-based measure has been validated in both community and experimental studies [31] and utilised in several randomised controlled trials to evaluate the effectiveness of physical activity promotion strategies [32,33], including several high-quality exercise referral scheme evaluations [10-12,14]. The 7-DAY PAR was administered by telephone interview. For those respondents who were contacted by telephone but refused to complete the time-consuming 7-DAY PAR telephone questionnaire, the GPPAQ was administered to give an indication of activity level at 12 month follow up.

The Baecke Questionnaire of Habitual Physical Activity (Baecke) [34] was designed for the measurement of habitual physical activity in epidemiological studies [35] and provides work, leisure, sport, and total activity indices. Each score can vary from 1-5, and the total score from 3-15, where a higher number indicates more physical activity. Baecke was included in the 12-month postal questionnaire.

##### - Anxiety and depression

The Hospital Anxiety and Depression Scale (HADS) [36] was used to assess depression and anxiety at 12 months. Scores on each of the two scales can range from 0-21, with higher scores indicating greater levels of anxiety or depression. HADS has been used extensively and has performed well in a variety of populations: A review of 747 studies concluded that it performed well in assessing the severity and presence of anxiety disorders and depression in both somatic and psychiatric cases in primary care patients and the general population [37].

#### Cost utility at 6 and 12 months

The primary outcome measure for the economic analysis will be the quality adjusted life year (QALY). This is a measure of health utility. This is calculated by 'weighting' each period of follow-up time by the value corresponding to the health-related quality of life reported by study participants [38,39]. Health related quality of life was assessed using the EQ-5D questionnaire collected at 6 and 12 months [40]. The EQ-5D is a validated generic health-related preference-based measure made up of five items covering mobility, self-care, usual activity, pain, anxiety and depression, each with three levels of severity (no problems, some problems, a lot of problems). EQ-5D is supported by National Institute for Health and Clinical Excellence (NICE). The Short Form 12 Health Survey (SF-12) assessed general health status at 6 and 12 months in terms of physical and mental functioning [41].

#### 6 month intermediate measures for exploratory analysis

It has been argued that evaluations of exercise interventions could usefully assess individual differences in responses to identify why particular intervention content may or may not be successful with particular groups or individuals and to generate theories of causation [42]. A number of measures were collected at 6 months for exploratory analysis.

##### - Motivation to exercise

This was assessed via the Behavioural regulation in exercise questionnaire-2 (BREQ-2) [43]. Based on self-determination theory [44] it posits that as motivation becomes more internalized the more it becomes the basis for autonomous behaviour. Thus, it might be expected that participants who start by being externally motivated but who become more internally motivated will have higher rates of adherence than those who, for instance, remain externally motivated. Previous studies have found it was predictive of adherence to exercise on prescription schemes [45]. The measure provides indices for levels of motivation, external, introjected, indentified, and intrinsic motivation, as well as a relative autonomy index (RAI). Scores on each index can range from 0-4, and between -24 - 20 for the RAI, where higher scores indicate a higher degree of motivation.

##### - Self-efficacy

The self-efficacy and exercise habits survey [46] was used to measure perceived self-efficacy in terms of exercise behaviours. The two indices are 'sticking to it' and 'making time for exercise'. Each can vary between 1 - 5, where a higher number indicates greater self-efficacy. It has been shown that high perceived self-efficacy facilitates goal-setting, effort investment, persistence in face of barriers and recovery from setbacks [47]. In a review of physical activity studies, Lewis et al. [48] suggest there is some support for self-efficacy as a mediator of physical activity.

##### - Social support

The social support and exercise survey 1987 [49], provides three indices for family social support, friends social support, and family rewards and punishments. Scores on the first two can vary from 5-50, where higher numbers indicate better quantity, structure, and or content of social relationships which facilitate exercise behaviour. Scores on the final area can vary between 2-15, where higher numbers indicate more punishment for exercising. Social support has been linked to a number of health outcomes including adherence to medical regimens, success in smoking cessation, adherence to exercise and enhanced weight loss treatment, although findings have not always been consistent [48,49].

#### Variables from programme database

##### - Programme attendance

A variable measuring programme attendance for each participant was defined using programme attendance data recorded by the EP. This variable has three levels: those who have received no intervention, a partial intervention (between one and 15 weeks), or the full intervention of 16 weeks.

##### - Programme implementation fidelity

Scores for fidelity to protocols are calculated for each of the 12 areas by allocating a score of 1 each time for; an instance of 4 week contact being made with patients who have stopped attending at this point, for follow up consultations being conducted at 8 months, and for consultations at 12 months with those completing the programme. A score of 0 was allocated for each instance of non contact, providing a range of 0 to 3 and allowing calculation of percentage fidelity overall and by area/exercise professional. An additional measure of fidelity is the quality of goal setting, with goal records coded as measurable and time bound (1) or as not measurable and time bound (0), allowing calculation of percentage fidelity overall and by area/exercise professional.

### Data collection

The research team were responsible for distributing, collecting and processing postal questionnaires at baseline, 6 months and 12 months. Respondents completed an initial informed consent form and subsequent mailings highlighted that they were free to withdraw from the study at any point. Questionnaires were anonymised, featured a unique ID and were accompanied by a freepost return envelope. Questionnaire non-responders were sent a repeat mailing two weeks after the first. For the primary outcome at 12 months, a specialist health research call centre team based at Cardiff University were employed to conduct telephone interviews using a standardised protocol [50] and blind to condition. Researchers again highlighted that respondents were free to withdraw from the study at any point. All telephone interviewers were trained and monitored to maximise standardisation of interviews. A strict protocol was adhered to in all cases, with each participant dialled on at least 15 occasions at different times and days of the week before being categorised as a non-respondent. Responders to 6 and 12 month questionnaires and 12 month telephone interviews were offered the opportunity to enter separate free prize draws for prizes of £200, £100 and £50 each time.

### Sample size

The trial sample size was determined to detect a difference in the primary outcome, total minutes of weekly exercise at 12 months. The planned sample size of 1052 participants in each group, has 90% power to detect an effect size of 0.15 with no loss to follow-up, and, more realistically, 84% and 87% power to detect an effect size of 0.15 if 25% and 20% respectively of randomised participants are lost to follow up.

### Data analysis

The primary analysis of the primary outcome is a regression model with the stratification variables (gender/LHB area), age group (16-44, 45-59, 60+) and baseline activity level (GPPAQ) included as covariates. In the event that the primary outcome has a highly skewed or bimodal distribution, it will be recoded as a five level ordinal variable, and ordinal regression used in the primary and secondary analyses. These analyses will be conducted on an intention-to-treat basis, in which each participant is coded according to the treatment condition to which they were randomised. A secondary analysis of the primary outcome repeats the primary analysis, but excluding baseline activity level as a covariate. For the primary outcome only, the primary and secondary analyses will be conducted (i) for all participants completing the 7-DAY PAR and (ii) with the addition of multiply imputed values of the primary outcome for those who did not complete the 7-DAY PAR but who did complete either the Baecke or GPPAQ instruments at 12-month follow-up.

The primary analysis for the primary outcome, and for imputed values of 7-DAY PAR, is repeated to conduct sub-group analyses for each of the following variables: gender, age group (16-44, 45-59, 60+), referral reason (mental health only, CHD only, or combination of CHD and mental health), Welsh Index of Multiple Deprivation tertile, fidelity of programme implementation in LHB area (high/low). In each case, the statistical significance of sub-group effects is assessed by including an interaction term in separate models for each respective sub-group variable, with each model also including the main effect for the respective sub-group variable. An analysis to identify whether outcomes vary in terms of exposure to the programme replaces the binary intervention group variable with the three level programme attendance variable.

The analyses described in the preceding two paragraphs are also conducted for the secondary outcome. However, the primary analysis for the secondary outcome (HADS anxiety and depression scales) is conducted only among participants referred for mental health only or a combination of mental health and CHD reasons. The analyses of the secondary outcome among all participants is secondary to this analysis.

Other analyses to be conducted, but not associated with formal hypothesis testing, will include an analysis of baseline demographic characteristics and their relationship with outcomes and the relationship between 6-month intermediate variables and the primary and secondary outcomes. This analysis will require complex multivariable models and mediation analyses estimated within a multilevel modelling framework to allow for independence in measures within individuals (over time) and within exercise specialist.

### Nested process evaluation

A nested process evaluation examined programme theory, programme implementation and how NERS was received, facilitating an in-depth understanding of the views of providers and users, as well as interpretation of outcome effects [51]. Guided by the framework proposed by Steckler and Linnan [52], the process evaluation explored diffusion of the national policy across local contexts, fidelity and dose of implementation, patient experiences, programme reach and recruitment. The process evaluation adopted a pluralistic multi method approach involving triangulation [53] of multiple perspectives and methods (Table 2). All analysis will be conducted prior to any knowledge of outcome effects to guard against interpretation bias

**Table 2:** Process evaluation methods

Participants	Methods	Areas for investigation
Welsh Assembly Government representatives (n = 3)	Group interviews and email/telephone communication	Programme theory Programme diffusion and barriers and facilitators to implementation

General Practices (n = 9)	Group interviews with primary care clinicians (GPs, practice nurses; practice managers)	Recruitment Uptake and reach

All exercise co-ordinators (n = 12)	One to one telephone interviews	Fidelity and dose Programme diffusion and barriers and facilitators to implementation Recruitment Uptake and reach Trial experience and implementation

All exercise professionals (n = 38)	One to one telephone interviews	Fidelity and dose Patient experiences - perceived impact and processes of change Uptake and reach Trial experience and implementation

Patient interviews (n = 32 within 6 centres)	Group and one to one interviews with patients at varying stages of the scheme	Patient experiences - perceived impact and processes of change

Exercise professionals (n = 23)	Recordings of at least one first consultation for all exercise professional and coding using Behaviour Change Counselling Index (BECCI; (Lane, Huws-Thomas et al. 2005).	Fidelity

Routine monitoring data (1080 patients)	Secondary analyses	Fidelity and dose Uptake and reach

In order to elicit programme theory and provide a framework for implementation checks, a programme logic model was developed through examination of scheme protocols and telephone and email contact with policy representatives. Quantitative assessments of programme implementation in terms of fidelity (i.e. consistency with programme theory) and dose delivered, draws on a combination of self reports from professionals and coordinator telephone interviews, as well as routine monitoring data and structured observation of tape recorded consultations. Exercise professionals were supplied with recording equipment and asked to provide recordings for at least one initial consultation, rated by two independent coders for fidelity to motivational interviewing principles using the Behaviour Change Counselling Index (BECCI); [54]. Programme uptake and reach are quantitatively assessed using routine monitoring data.

Experiences of diffusing the national policy into local contexts were explored through qualitative semi-structured telephone interviews with policy representatives and all local area coordinators. All exercise professionals were invited to participate in one to one telephone interviews exploring issues including experiences of implementing the scheme and perceived patient responses to the programme. In order to incorporate patients' perspectives on the programme, six case study centres were sampled purposively to represent a range of geographical areas and levels of deprivation and two members of the research team visited each centre. Patients attending the centre on the day of the visit were informed in advance of the researchers' attendance, and were invited to participate in group or one to one semi-structured interviews immediately after classes finished. In addition, GPs, practice nurses and practice managers referring patients to the scheme were interviewed, exploring influences on trial and scheme recruitment. Nine group interviews were conducted across areas, practice size and level of referral.

### Nested economic evaluation

The nested economic evaluation [55-57] took a public sector, multi agency perspective, spanning the Welsh Assembly Government (WAG), local government and the NHS. It costed the national exercise referral programme using WAG and local authority exercise programme budgets. In addition, telephone interviews with all principal leisure centre managers ascertained any additional expenditure incurred by local authorities in establishing and running NERS. Similarly telephone interviews ascertained the costs to WAG of establishing and coordinating the Scheme. The questionnaire data recorded trial participant primary and secondary care health service use, costed using national unit costs [58,59]. With this, we will conduct a primary cost utility analysis to calculate the cost per QALY of NERS. We will undertake sensitivity analysis to explore what happens when we add in the cost of a specific GP consultation to facilitate referral onto NERS, and the effect of participants paying £1 or £2 per exercise session. Sensitivity analysis will be particularly important in the application of economic evaluation methods to a large pragmatic public health trial [60]. We will undertake subgroup analysis to investigate how age, gender, adherence and main reason for referral e.g. CHD risk or mental health problems, affects our estimates of the cost per QALY of NERS. We will use EQ-5D as our measure of utility to generate a cost per QALY and Cost Effectiveness Acceptability Curve (CEAC) for comparison with the NICE ceiling of £30,000 [61].

### Recruitment

Trial recruitment occurred in 12 of the 13 areas, with one area failing to start a scheme during the trial period. Between July 2007 and October 2008, 4,779 health professional referrals were received by the evaluation team. Of these, 1,493 were not eligible for the trial and their referral forms were forwarded to the ECs for processing directly onto the scheme. The remaining 3,286 were sent full informed consent information and a baseline questionnaire. In total 2,160 patients were recruited to the trial, with 890 not responding and 236 not consenting to participate in the trial.

Respondents ranged between 16 and 88 years old, with a mean age of 52 (SD 14.7) and the vast majority classed themselves as white (96%). Whilst, measures of area deprivation ranged from 2.3 to 81.0 with a mean of 22.6 (SD 14.6). Table 3 shows that the majority were referred for CHD risk factors on their own (72%) or in combination with mental health conditions (24%), with only 4% referred solely for mental health reasons. Those recruited were most likely to be female (66%), to be married or with a partner (61%) and there was a fairly equal split between those employed (31%) and retired (32%). Finally, whilst the majority referred onto the scheme classified themselves as either inactive or moderately inactive (74%), 24% defined themselves as either active or moderately active.

**Table 3:** Baseline measures for recruited sample

Measure	% (N)
**Reasons for referral** CHD CHD and Mental Health Mental Health	72.2 (1,559) 24.2 (522) 3.7 (79)

**Gender** Female Male	65.5 (1,415) 34.5 (745)
**GPPAQ** Inactive Moderately inactive Moderately active Active Missing	58.6 (1,266) 15.3 (330) 15.8 (342) 8.2 (176) 2.1 (46)
**Employment** Retired Employed Housework Other Seeking work Student Missing	31.9 (666) 30.8 (646) 19.1 (413) 13.1 (282) 3.2 (69) 1.9 (40) 2.0 (34)
**Education** > Minimum education	52.1 (1,127)
**Marital status** Married/Partner Single Divorced/Separated Widowed Missing	61.4 (1,326) 17.8 (384) 13.6 (293) 6.0 (129) 1.3 (28)

## Discussion

Previous attempts to facilitate rigorous evaluations of exercise referral schemes have been hampered by policy and practice constraints [25,26]. Existing studies meanwhile, have suffered from a number of methodological shortcomings, not least significant barriers to recruitment [6] and a lack of understanding of the causal and implementation process of ERS means that we still do not know what types of ERS have worked, why, for whom, in what circumstances and at what cost [28]. To address these shortcomings, this study included early and sustained collaboration with policy and practice partners, adopted an innovative approach to trial recruitment and included comprehensive nested process and economic evaluations. Such strategies will not only facilitate rigorous evidence on the effectiveness of the national exercise referral scheme but provide examples of good practice for the design and conduct of trials of similar policy interventions.

### Collaboration with policy and practice partners

The difficulties in facilitating rigorous evaluations of policy interventions is well recognised, particularly for ERS [25,26]. This evaluation included a substantial pre trial development phase. Early engagement between evaluators, the national policy co-coordinator (EM) and local implementers at the intervention development and implementation stage meant that a rigorous design could be negotiated that met both scientific and practical considerations. Regular meetings with policy makers and practitioners also ensured that the research design was understood and maintained. The involvement of government social researchers (JH, CR) who promote RCTs of policy evaluations with ministers and support policy colleagues when they are implemented was key to the success of this process. Such an approach is enshrined in the work of the Public Health Improvement Research Network (PHIRN) in Wales which develops and supports multidisciplinary research groups of academics, policy makers and practitioners to identify and develop opportunities for rigorous policy evaluations.

Collaboration with national policy leads and local practitioners was also key in developing and implementing programme monitoring systems. The provision of a national, rather than a number of local schemes, offered the opportunity to introduce a standardised national monitoring system. This included the provision of a common database and protocols for data collection and assessment. High quality programme monitoring data can provide important information, such as referral and completion rates and useful data on programme fidelity. The evaluation will use this information to assess the impact of adherence and fidelity on effectiveness Robust programme monitoring systems also provide the opportunity for long term self evaluation once trials such as this are completed.

### Establishing effective recruitment strategies

In Wales, the NERS was introduced within a RCT design in the context of the termination of pre-existing local schemes. This was particularly challenging, as a significant barrier to facilitating rigorous designs has been professional reluctance to refer into trials when there is a perception of change or withdrawal of service, regardless of the evidence base for the effectiveness of that service [25]. Establishing effective recruitment strategies was further complicated by a reliance on primary care clinicians as the main source of referral into the scheme. It has been argued that such clinicians are well placed to promote increased physical activity [62,63]. However, previous studies have found that physical activity promotion was not a priority during routine consultations [42,64], that there is a lack of consensus in how clinicians perceive their role in changing patients' behaviour and that patients are frequently referred in an unsystematic way [42,65].

Despite this, the trial achieved referrals from 12 of the 13 implementation areas (with one area failing to start the scheme) and a high referral rate from clinicians. This may be in part a function of the size of the scheme being evaluated, but was also facilitated by a number of strategies employed in the conduct of this study. This included briefing sessions for professional stakeholders by the evaluation and national policy team at an early stage to address concerns and promote the evaluation. At the local level, the provision of Exercise Coordinators who were able to promote the trial with clinicians also facilitated professional participation. Particularly important was the fact that trial recruitment was undertaken by the evaluation team after clinicians had provided basic information to patients. This placed a low research burden on health professionals who promoted but did not recruit to the trial.

Examining characteristics of those recruited to the trial highlights the greatest proportion were referred for CHD risk, that there were a larger number of females and a significant minority who classified themselves as active or moderately active. Although the scheme was targeted at sedentary individuals, this latter finding reflects the pragmatic nature of the trial which placed minimal control on the implementation of the scheme other than randomisation. Results are therefore likely to be highly reproducible and have high external validity. This is particularly important given previous criticisms of RCTs of ERS that what is gained in internal validity is often at the expense of external validity [66]. Influences on NERS referral processes and the acceptability of trial recruitment strategies will be assessed within the nested process evaluation which will explore influences on clinician referral such as lack of time during consultations; knowledge about ERS, views on evaluation; and medico- legal responsibility [42,67-69].

### A comprehensive understanding of the intervention

Good quality evaluative research not only quantifies outcomes, but also helps us to understand how an intervention produces the outcomes that it does [70]. This is challenging for the evaluation of ERS which are typically heterogeneous and driven by differing latent programme theories, which can only realistically be compared with other similarly conceived schemes [71]. However, no previous evaluations have clearly articulated ERS programme theories and attempted to establish congruence between the conceived and the delivered intervention. In this study, comprehensive nested process and economic evaluations will attempt to address these shortcomings. An intervention logic model will be developed through discussions with implementers which will identify key proposed programme inputs and the anticipated intermediate outcomes associated with these inputs. This will provide the basis for a comprehensive assessment of intervention implementation and will inform attempts to map causal pathways.

Programme implementation will be assessed initially by case studies of community sports centres, purposively sampled to reflect a range of area level socioeconomic deprivation and geographic regions. These will examine intervention delivery across contexts [27] and explore participant experiences of NERS [72-74]. Data will also inform the development of structured interview schedules for national and local implementers to assess aspects of implementation such as diffusion, reach and fidelity. Aspects of fidelity will also be assessed via routine monitoring data records and systematic checks of treatment integrity [75]. This follows recommends that the delivery of motivational interviewing should be assessed through coding of tape recorded consultations, rather than relying upon self reports of the delivering party [76]. Finally, complex interventions also need to be understood as leading to outcomes through the activation of mechanisms of change, with the activation of these mechanisms varying across contexts and between subgroups [77]. This study has therefore includes a number of variables assessed at 6 months that will be used to model causal processes amongst a number of sub groups. Taken together with the nested economic evaluation these elements provide the opportunity not only to judge the effectiveness of the NERS, but to understand why it may be effective, for whom, under what circumstances and at what cost [28].

## List of abbreviations

7D-PAR: Seven Day Physical Activity Recall interview; BECCI: Behaviour Change Counselling Index; BREQ-2: Behavioural Regulation in Exercise Questionnaire-2; CEAC: Cost Effectiveness Acceptability Curve; CHD: Coronary Heart Disease; EC: Exercise Co-ordinator; EP: Exercise Professional; ERS: Exercise Referral Schemes; GP: General Practitioner; GPPAQ: General Practice Physical Activity Questionnaire; HADS: Hospital Anxiety and Depression Scales; LHB: Local Health Board; NERS: National Exercise Referral Scheme; PAR: Physical Activity Recall; QALY: Quality Adjusted Life Year; RCT: Randomised Controlled Trial; SF-12: Short Form 12 Health Survey; WAG: Welsh Assembly Government

## Competing interests

The authors declare that they have no competing interests.

## Authors' contributions

Principal responsibility for the main study design is assumed by SM. LR is the trial manager and responsible for the day to day running of the study. GM is responsible for the development and day-to-day running of the mixed-method process evaluation. RTE is responsible for the economic evaluation study design. PL is responsible for the day to day running of the economic evaluation. NW contributed to the study design and aspects of the process evaluation. JH and CR were responsible for developing the initial research specification and for input into the study design and EH for facilitating the implementation of the research design. LM contributed to the study design, conducted sample size calculations and designed the analysis plan. LR produced a first draft of the manuscript and SM was responsible for the final revised version developing and integrating contributions from GM, RTE, PL, NW and LM. All authors read and commented on drafts and approved the final manuscript.

## Pre-publication history

The pre-publication history for this paper can be accessed here:

http://www.biomedcentral.com/1471-2458/10/352/prepub
